# Epithelial-Mesenchymal Transition Regulated by EphA2 Contributes to Vasculogenic Mimicry Formation of Head and Neck Squamous Cell Carcinoma

**DOI:** 10.1155/2014/803914

**Published:** 2014-04-17

**Authors:** Wei Wang, Peng Lin, Baocun Sun, Shiwu Zhang, Wenjuan Cai, Chunrong Han, Li Li, Honghua Lu, Xiulan Zhao

**Affiliations:** ^1^Department of Otorhinolaryngology Head and Neck, Institute of Otorhinolaryngology, Tianjin First Central Hospital, Tianjin 300192, China; ^2^Department of Pathology, Tianjin Cancer Hospital, Tianjin Medical University, Tianjin 300060, China; ^3^Department of Pathology, Tianjin Medical University, Tianjin 300070, China; ^4^Department of Pathology, University of Texas, MD Anderson Cancer Center, Houston, TX 77030, USA; ^5^Department of Pathology, Tianjin First Central Hospital, Tianjin 300192, China

## Abstract

*Purpose*. Vasculogenic mimicry (VM) was related to invasion and metastasis of head and neck squamous cell carcinoma (HNSCC) patients. This study was designed to investigate the role of EphA2 in VM formation of HNSCC. *Methods*. The SiRNA technique was used to knock down the expression of EphA2 *in vitro*. The ability of cell migration and invasion were measured by transwell and wound healing assays; three-dimensional culture was used to detect the ability of channel-like structure formation; Western blot was used to detect the expression of epithelial-mesenchymal transition- (EMT-) related molecules *in vitro*. Further semiquantitative real-time RT-PCR assays and immunohistochemistry were used to demonstrate expression of EphA2 and EMT-related molecules according to VM presence or not in human tissue. *Results*. Knocking down EphA2 *in vitro* leads to disabled channel-like structure formation, reduction of invasion and migration ability, and reverse of EMT-related markers. Both semiquantitative real-time RT-PCR and immunohistochemistry showed that expressions of EphA2, Twist, and Vimentin were higher in the VM-positive group than in the VM-negative group significantly, while expressions of E-cadherin, claudin4, and DSG-3 were reverse. *Conclusions*. EphA2 played a key role in VM formation of HNSCC through regulation of EMT.

## 1. Introduction

Head and neck squamous cell carcinoma (HNSCC) is the second main upper respiratory tract tumor following lung cancer by incidence and mortality. The overall survival rate has remained unchanged at approximately 35–70% over the past several decades despite advancements in diagnosis and treatment. It is mainly caused by uncontrolled recurrence and local lymph node metastasis [1]. Thus, there is a demand for the development of new therapeutic targets for HNSCC, taking advantage of the disease's unique qualities.

Traditionally, tumor invasion and metastasis are known to mainly depend on angiogenesis/vasculogenesis. However, the results of studies in HNSCC associating microvessel density and various clinicopathological parameters and/or outcome are still inconclusive [2]. Vasculogenic mimicry (VM) is a new type of blood supplement. It is independent of angiogenesis. VM was constructed by highly invasive and genetically dysregulated tumor cells with a pluripotent embryonic-like genotype [3]. Such tumor cells facilitate plasticity to gain the capability to participate in neovascularization processes combined with extracellular matrix remodeling. Ultimately, a fluid-conducting, matrix-rich meshwork is constructed [4]. It has been earlier described in some mesenchymal tumors [5] and now spread to epithelial carcinomas [4, 6]. VM in synoviosarcoma, rhabdomyosarcoma, and hepatocellular carcinoma has been reported by the authors' laboratory and collaborators in recent years [7, 8]. Tumors that exhibit VM are related to more aggressive biology and increased tumor-related mortality [9]. We previously identified that VM existed in laryngeal squamous cell carcinoma (LSCC) and contributed to the progression by promoting lymph node metastasis, which was an independent predictor of a poor prognosis of LSCC [10]. However, the pathway through which VM impacts tumor biology remains unclear. Therefore, it is necessary to declare the important biomarkers of VM in HNSCC.

EphA2, an embryonic phenotype, has been confirmed as a key factor promoting VM formation through cell plasticity [11] in melanoma. Wang et al. [12] have demonstrated its application in ovarian cancer by interaction with VEGF-a. However, roles of VM might be variable in different tumors. And whether it plays a specific role in HNSCC remains to be investigated.

In this study, we detected existence of VM in human tissue of HNSCC, as well as in a three-dimensional cell culture. Moreover, further* in vitro* study demonstrated that EphA2 played a key role in VM formation of HNSCC through regulating epithelial-mesenchymal transition (EMT). It may be a potential target molecule for HNSCC therapy in the future.

## 2. Materials and Methods

### 2.1. Cell Lines, Culture Conditions, and Reagents

Three human head and neck squamous cell carcinoma (HNSCC) cell lines, Hep-2 (larynx), Tb (tongue), and CNE-2 (nasopharyngeal), were utilized. Hep-2 was obtained from the preclinical medicine cell centre of China Union Medical College. Meanwhile, Tb was presented by the Ninth Peoples' Hospital of Shanghai, and CNE-2 was provided by the Immunology and Biotherapy Laboratory of Tianjin Cancer Hospital. Cells were grown as monolayer in RPMI 1640 supplemented with 10% FBS (Invitrogen) and 0.1% gentamicin sulfate (Invitrogen) at 37°C in a humidified 5% CO_2_ atmosphere. The recombinant human epithelial growth factor (EGF) was obtained from R&D Systems (Minneapolis, MN, USA). Alexa 568-phalloidin was attained from Molecular Probes, Inc.

### 2.2. RNA Interference

Following the manufacturer's instructions, transfection was performed by using Lipofectamine 2000 (Invitrogen) with EphA2-specific siRNA (5′-AATGACATGCCGATCTACATG-3′) and the scramble sequence siRNA (GeneChem, Shanghai, China). Meanwhile, the siRNA construct containing a scrambled sequence was transfected into the cells to generate control cells. The transfected cells were selected on the basis of their resistance to Hygromycin B (BD Biosciences, San Jose, CA, USA). Rescue experiments were carried out for migration, invasion, chemotaxis, and Western blotting analysis. The expression of EphA2 protein was monitored by Western blotting to establish cell lines stably expressed by the mutant proteins.

### 2.3. Western Blotting Analysis

Western blotting analysis was performed as previously described [13]. The antibodies against EphA2, E-cadherin, claudin4, DSG-3, Twist, and Vimentin were acquired from Santa Cruz Biotechnology, Inc.

### 2.4. Three-Dimensional Cultures

A total of 100 microliters of Matrigel (Invitrogen) and 100 microliters of cells (5 × 10^6^/mL) were dropped into a 24-well plate and allowed to polymerize for 1 hr at 37°C. Another 100 microliters of culture fluid was added to the well, blended, and incubated for 10 days. Culture fluid was replaced every two days. The culture lasted for about 30 days when cells began to die.

### 2.5. Wound Healing Assay and Invasion Assay

Wound healing assay was performed as previously described by Guo et al. [14]. The speed of wound closure was monitored by phase-contrast microscopy at 0, 3, 6, 9, 12, and 24 h time points. Chemotaxis assay was performed in a 48-well Boyden chamber as described by Sun et al. [15]. A Boyden chamber invasion assay was performed as previously described by Albini et al. [16].

### 2.6. Patients and Tumor Samples

This study enlisted a total of 203 patients with histopathologically diagnosed HNSCC and treated at the Tianjin Cancer Hospital's Department of Head and Neck Surgery from January 1990 to January 2003. All the cases have complete data of clinical pathological and follow-up. The Otorhinolaryngology Head and Neck Department of Tianjin First Central Hospital offered fresh freezing tissues. The Tianjin Cancer Hospital's and the Tianjin First Central Hospital's ethics committee approved the study protocol.

### 2.7. Immunohistochemistry of Monostaining and Double Staining and Regents

Monostaining of EphA2, E-cadherin, claudin4, DSG-3, Twist, and Vimentin and double staining of CD31/periodic acid-Schiff (PAS) for VM were performed as previously described by Sun et al. [17].

The antibodies against EphA2, E-cadherin, claudin4, DSG-3, Twist, and Vimentin were acquired from Santa Cruz Biotechnology, Inc. CD31 was purchased from Zhongshan Golden Bridge Biotechnology Co. Ltd., Beijing, China. The 0.5% periodic acid and Schiff solutions were prepared in Department of Pathology in Tianjin Cancer Hospital.

### 2.8. Evaluation of the Staining

VM was roughly identified using hematoxylin-eosin staining slides. By subsequent double staining, it can be identified as PAS-positive loops surrounded by tumor cells (not endothelial cells), with or without red blood cells. The above 203 cases of head and neck squamous cell cancer were subsequently divided into two groups: VM positive (43 cases) and VM negative (160 cases). The semiqualitative method recorded by the staining index (SI) [17] was employed to investigate the expression of EphA2, E-cadherin, claudin4, DSG-3, Twist, and Vimentin. The positive index (PI) was employed to evaluate the expression of Twist and Vimentin.

### 2.9. Quantitative** **Real-Time**  **Revers** **Transcription-Polymerase Chain Reaction Analysis

The real-time RT-PCR analysis was done as described [18]. The results were expressed as n-fold differences in target gene expression relative to the GAPDH, which represents the relative expression value of each sample's targeted gene. The primers of forward and reverse were exhibited below: EphA2 5′*-CCG CAA CAT CCT CGT CAA C-*3′*; *5′*-ACA ATG CCA AAG CTC CAC ACG TC-*3′*; *E-cadherin 5′*-GTG GTC AAA GAG CCC TTA CT-*3′*; *5′*-TGG TGC AAC GTC GTT ACG AG-*3′*; *claudin4 5′*-AGG CCA AGA CCA TGA TCG T-*3′*; *5′*-CCA CCA GCG GAT TGT AGA AG-*3′*;* DSG-35′*-GAT AAT GAA GGC GCA GAT-*3′*; *5′*-CCA TAA CCG CTG TCT TTA GAG G-*3′*;* Twist 5′*-ACA CTA GGC CAC GCA TCT-*3′*; *5′*-CTC AGC ATA CCC AAT AGG CA-*3′*;* Vimentin 5′*-GGG AGT CCG CAG TCT TAC GA-*3′*; *5′*-TCC AGA CCG AGA AGG CGT AG-*3′*; *GAPDH 5′*-AGATCCACAACGGATACATT-*3′*; *5′*-TATGACAACTCCCTCAAGAT -*3′.

### 2.10. Statistical Analysis

All analyses were performed using the SPSS software (v.15.0, Chicago, IL). Statistical significance was set at *P* < 0.05. All the results were obtained from at least three separate experiments.

## 3. Results

### 3.1. Identification of VM

**Figure 1:**
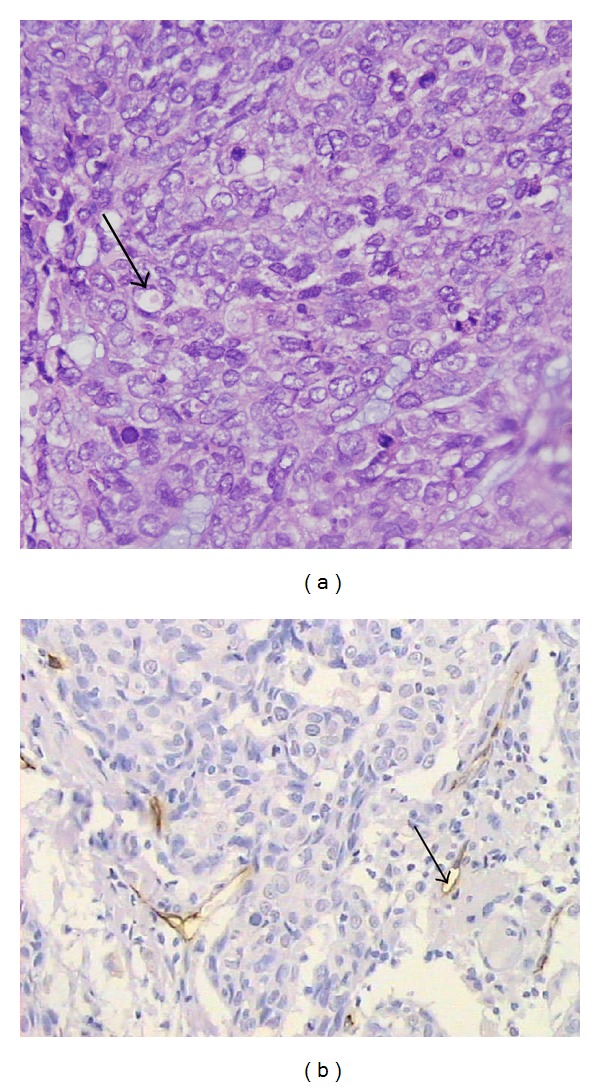
Identifying VM and EDV by PAS and CD31 double staining ((a), (b)). (a) The VM channel (black arrow) in human sample is formed by head and neck squamous carcinoma cells. There are red blood cells in the center of the channel. PAS-positive substances line the channel and form a basement membrane-like structure (pink) (magnification: ×400). (b) Endothelium-dependent vessels (black arrows) are lined by spindle-shape endothelial cells, which are stained by CD31 (brown). The vessels' basement membrane is positive for PAS staining (pink) (magnification: ×200).

**Figure 2:**
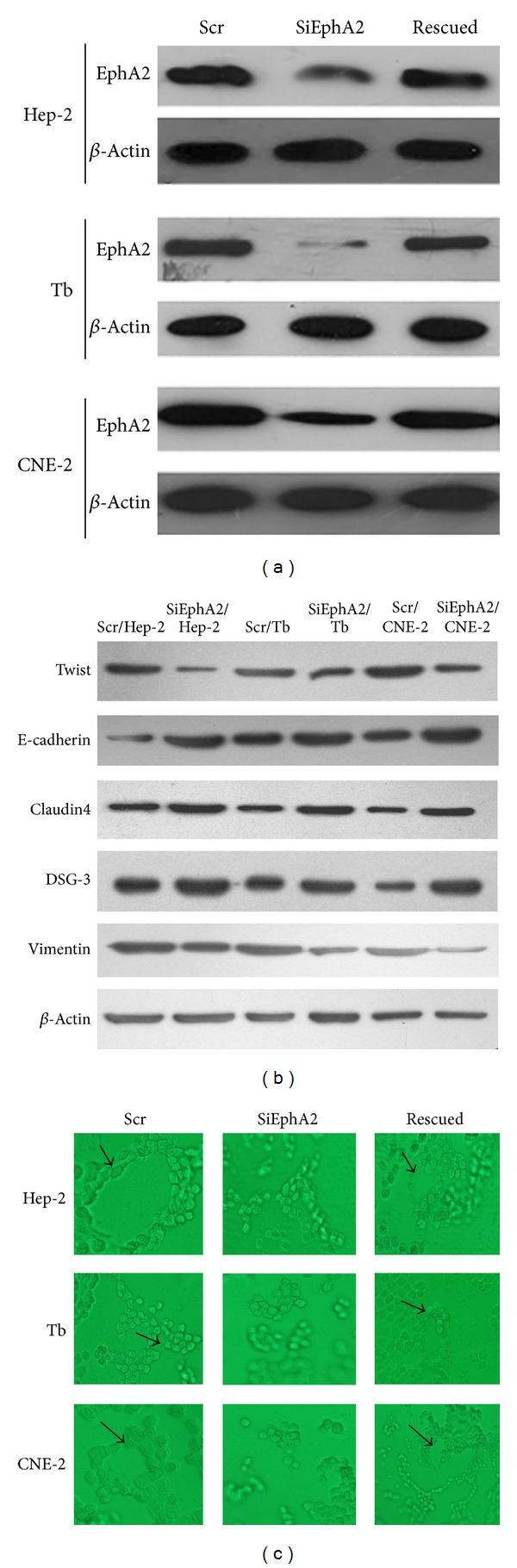
Three-dimension cell culture in three HNSCC cell lines (Hep-2, Tb, and CNE-2) ((a), (b), and (c)). (a) The protein expression of EphA2 was inhibited by siRNA and retrieved after a sequence of overexpression of EphA2 was transfected into siEphA2 cells, which were monitored by Western blotting. (b) Disruption of EphA2 by siRNA leading to the change expression of EMT-related molecules measured by Western blotting. There was a decreasing expression of Twist and Vimentin and an increasing expression of E-cadherin, claudin4, and DSG-3 compared with the control cells (*P* < 0.05). (c) Hep-2, Tb, and CNE-2 cell lines can construct channel-like structures in Matrigel. All cell lines lost the capability to form channel-like structures in Matrigel after EphA2 was knocked down compared with the control cells. And regaining of EphA2 expression reconstructed channel-like networks formation in three cell lines. The black arrows represent the cross-section of the tubular networks, indicating that they contain hollow lumen-like structures (original magnification: ×400).

VM existed in HNSCC (Figure 1(a)). It showed that VM was formed by tumor cells, but not endothelial cells, without hemorrhage, necrosis, or inflammatory cells infiltrating these structures. VM was identified through the detection of PAS-positive loops surrounding tumor cells (not endothelial cells), with or without red blood cells. In CD31-stained slides, there were no positive cells in VM. Recent discoveries in the field of melanoma and ovarian cancer research have suggested that the vasculogenic-like patterned networks formed by tumor cells* in vitro* may account for a subcategory of highly viable, nonangiogenic tumors seen* in vivo* [3, 4]. The endothelium-dependent vessel showed a CD31-positive endothelial cell to form the vessel wall (Figure 1(b)). We also performed three-dimensional cell culture in Matrigel in three HNSCC cell lines, Hep-2, Tb, and CNE-2 cells, to assess their ability to form vasculogenic structure* in vitro*. All of them were able to invariably form vasculogenic-like tube structure. Microscopic observation of the network evolution revealed steady outgrowths which developed into tubular patterns interconnecting spheroidal nests of cells (Figure 2(c)).

### 3.2. Downregulating the Expression of EphA2 Impaired Channel-Like Structure Formation and the Upregulation of EphA2 Retrieved Its Ability

EphA2 is a transmembrane tyrosine kinase receptor involved in signal-transduction pathways that functions in the regulation of cellular adhesion, migration, and invasion [11, 19]. Overexpression of EphA2 had been observed in many human cancers. The expression of EphA2 in three HNSCC cell lines, Hep-2, Tb, and CNE-2 cells, was first examined by Western blotting. SiRNA technology was applied to inhibit EphA2 expression. A scrambled sequence of siRNA was transfected into the cells to generate control cells, which were designated as scr/HNSCC cells. Later construction of cells overexpressed EphA2 was to build rescued group. Transfected cells were screened for EphA2 expression using Western blotting analysis (Figure 2(a)). The cells were selected by Hygromycin B resistance to establish cell lines that stably downregulated or upregulated expression of EphA2. Following Hygromycin B selection, transfected cells were screened for EphA2 expression using Western blotting analysis. Almost 70–80% of EphA2 protein levels were reduced in siEphA2/HNSCC cells compared with those in scr/HNSCC cells. And the rescued cell appeared to reestablishment of EphA2 in all three cell lines.

To investigate the role of EphA2 in the formation of tubular networks, cells were cultured in Matrigel. Scr/HNSCC cells and rescued cells appeared to form channel-like structures around the 7th day. The presence of a network persisted for about 30 days. Then cells began to die and experiments were generally terminated. The siEphA2/HNSCC cells seemed to fail to construct channel-like structures during this period. Knocking down EphA2 in cell lines showed marked inability to form vasculogenic-like structure compared with control cells, while upregulating EphA2 leads cell lines to regain ability of channel-like networks formation (Figure 2(c)). We suggested that EphA2 may play a key role in VM formation in HNSCC cell lines.

### 3.3. Downregulating the Expression of EphA2 Impaired Migration and Invasion of HNSCC Cells and Upregulating EphA2 Regained Their Capacity of Migration and Invasion

**Figure 3:**
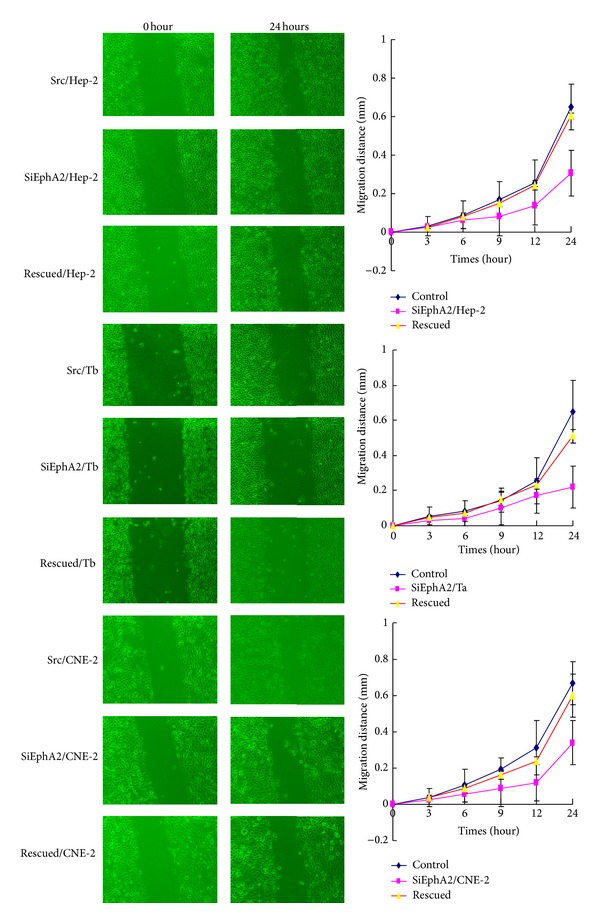
Wound healing assays in HNSCC cell lines. Three HNSCC cell lines knocking down EphA2 strongly reduced the capability of migration compared with the control cells and rescued cells (*P* < 0.05).

It has been recognized that VM was associated with tumor invasion and metastasis [9, 20]. EphA2 may be a regulator in VM formation. In order to examine how EphA2 affects cell migration and invasion, we performed wound healing assay and cell chemotaxis assay. Scratch assay, an* in vitro* wound healing assay, is a method for evaluating cell migration capacity. It was observed that scr/HNSCC cells and rescued cells migrated into the wound and resulted in wound narrowing within 24 h, whereas siEphA2/HNSCC cells were significantly less mobile (Figure 3) as supported by the delay in the mean distance of closure. All the cells were maintained in a medium supplemented with 0.5% FBS to block cell proliferation, which could otherwise account for gap closure. Taken together, our results obviously showed that EphA2 reduction by siRNA impaired the migration and that EphA2 was required for migration of HNSCC cells.

**Figure 4:**
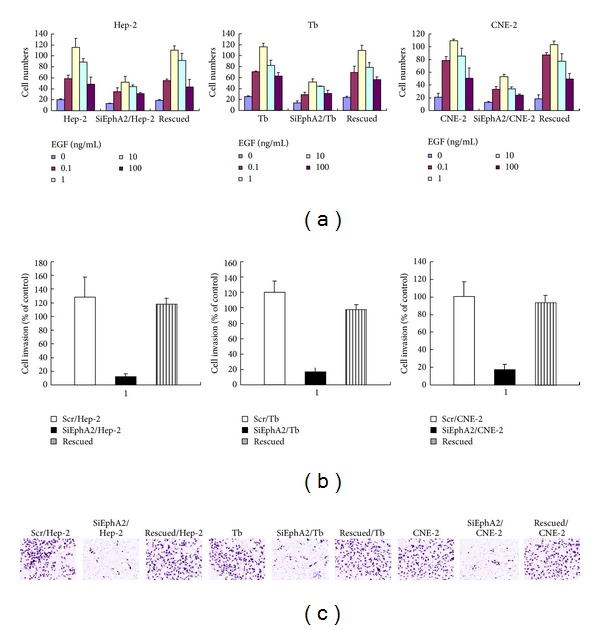
Chemotaxis and invasion assays in HNSCC cell lines. (a) SiEphA2/HNSCC cells, scr/HNSCC cells, and rescued cells induced by EGF appeared in a dose-dependent manner, although the chemotaxis of the siEphA2/HNSCC cells was impaired in response to EphA2 reduction. Chemotaxis assays showed that 1 ng/mL EGF was the optimization concentration for transwell. (b) Invasion assays. There was a remarkably reduced invasion in HNSCC cell lines compared with the control cells and rescued cells (*P* < 0.05).

Another method is chemotaxis referring to directional cell movement dependent on a concentration gradient. Robust chemotaxis of the siEphA2/HNSCC cells, scr/HNSCC cells, and rescued cells induced by EGF appeared in a dose-dependent manner, although the chemotaxis of the siEphA2/HNSCC cells was impaired in response to EphA2 reduction. These results revealed that EphA2 was required for HNSCC cell chemotaxis, while a concentration gradient was required for efficient migration in response to EGF (Figure 4(a)). Subsequently, Boden chamber invasion assays were utilized to confirm the three HNSCC lines' different invasive capabilities after EphA2 knockdown. Knockdown of EphA2 in cell lines significantly reduced the invasion capability compared with the control cells (Figure 4(b)), while overexpression of EphA2 retrieved the ability of invasion in all cell lines. Both results indicated that EphA2 knockdown may decrease migration and invasion capabilities of HNSCC cells. We speculated from our study that VM contributed to invasion and metastasis of HNSCC, and EphA2 may play a key role in this process.

### 3.4. Downregulating the Expression of EphA2 Reversed EMT

Similar to VM, EMT is another phenomenon of acquiring the capability of invasion and metastasis by changing into an embryonic genotype. Recently, it has been consistently demonstrated in epithelial malignancies [21, 22]. It is a type of plasticity during which epithelial cells lose many of their characteristics and acquire properties typical to mesenchymal cells. A key point of EMT is the reduction of cell-cell adhesion by transcriptional repression of cadherins (adherens junctions), occludin and claudin (tight junctions), and desmoplakin (desmosomes) [23–25]. The expression of intermediate filaments is also changing during EMT, such as Vimentin, being typical of mesenchymal cells. In addition, transcription factors, which induced EMT, also play a key role, such as Twist, Snail, Slug, SIP1/ZEB2 (Smad Interacting Protein)/(zinc finger E-box binding homeobox), deltaEF1/ZEB1, and the basic helix-loop-helix (HLH) transcription factor E47. To reveal the role of EphA2 during EMT, we inhibited EphA2 by siRNA to observe changing of EMT-related molecules in three HNSCC cell lines. It was showed that Twist and Vimentin decreased in protein expression, while epithelial adhesion markers E-cadherin, claudin 4, and DSG-3 increased compared with the control cells (Figure 2(b)). It is interesting to speculate that highly aggressive epithelial tumor cells may likewise overexpress the mesenchymal phenotype through the EMT procedure to facilitate VM formatting. EphA2 may impact VM formation through regulating EMT.

### 3.5. Relationship of VM and Expression of EphA2 and EMT-Related Molecules in HNSCC

**Figure 5:**
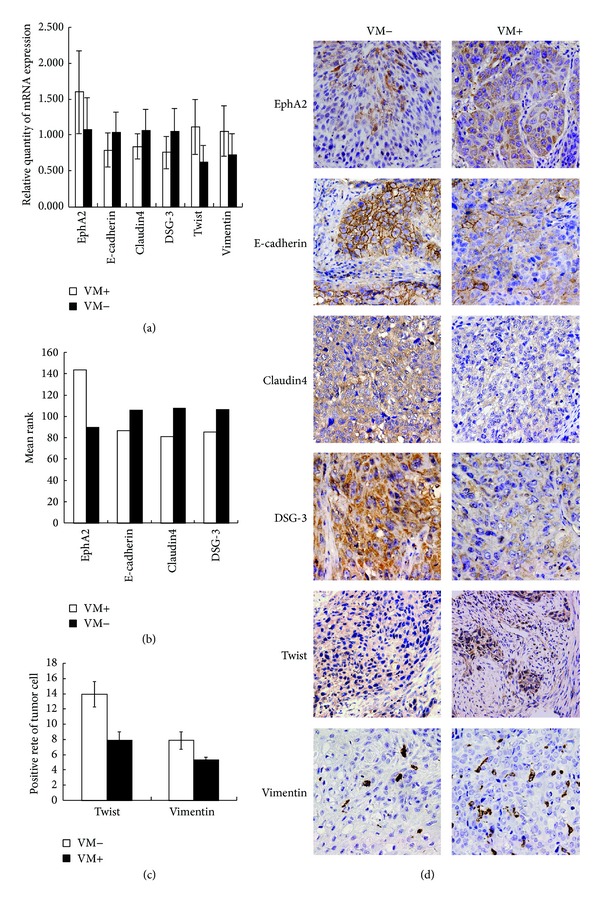
Different expressions of VM-related molecules in HNSCC patients between the VM-positive group and VM-negative group ((a), (b), (c), and (d)). (a) Different mRNA expressions of EphA2 (*P* = 0.023), E-cadherin (*P* = 0.035), claudin4 (*P* = 0.034), DSG-3 (*P* = 0.022), Twist (*P* = 0.001), and Vimentin (*P* = 0.024) between the VM-positive group and VM-negative group. (b) Different protein expressions of EphA2 (*P* = 0.004), E-cadherin (*P* = 0.044), claudin4 (*P* = 0.006), and DSG-3  (*P* = 0.032) between the VM-positive group and VM-negative group by immunohistochemistry. (c) Different protein expressions of Twist (*P* = 0.039) and Vimentin (*P* = 0.044) between the VM-positive group and VM-negative group by immunohistochemistry. (d) By immunohistochemistry, EphA2, E-cadherin, claudin4, DSG-3, and Vimentin were found to be expressed in cytoplasm of HNSCC cells. Twist was expressed mainly in the nucleus.

To further validate the above conclusion, we detected VM and expression of EphA2 and EMT-related molecules in human samples of HNSCC. Semiquantitative real-time RT-PCR was performed to detect correlated gene expression on 24 cases of frozen tumor tissue (12 VM-positive cases and 12 VM-negative cases). mRNA expression of EphA2 (*P* = 0.023), Twist (*P* = 0.001), and Vimentin (*P* = 0.024) was significantly higher in the VM-positive group than in the VM-negative one, while E-cadherin (*P* = 0.035), claudin4 (*P* = 0.034), and DSG-3 (*P* = 0.022) were significantly lower in the VM-positive group than in the VM-negative one (Figure 5(a)). Furthermore, by immunohistochemistry, EphA2, E-cadherin, claudin4, DSG-3, and Vimentin were found to be expressed in cytoplasm of HNSCC cells. Twist was expressed mainly in the nucleus (Figure 5(d)). The SI of EphA2  (*P* = 0.004) (Figure 5(b)) and PI of Twist (*P* = 0.039) and Vimentin (*P* = 0.044) (Figure 5(c)) were higher in the VM-positive group than in the VM-negative one, while the SI of E-cadherin (*P* = 0.044) (Figure 5(b)), claudin4 (*P* = 0.006), and DSG-3  (*P* = 0.032) were significantly lower in the VM-positive group than in the VM-negative one (Figure 5(c)). We deduced from our study that expressions of EphA2 and EMT-related molecules are associated with VM formation in HNSCC.

## 4. Discussion

VM refers to the* de novo *generation of tumor microcirculation without participation of endothelial cells. It was first reported in melanoma by Maniotis in 1999 [3]. Previous research has demonstrated existence of VM in most mesenchymal tumors. We first identified that VM existed in squamous cell carcinoma to disclose the secret why we fail to explain invasion and metastasis only by angiogenesis and being inefficient in antiangiogenesis therapy for HNSCC clinically [10]. In this study, we further illustrated that EMT regulated by EphA2 contributed to VM formation in HNSCC. It elucidated the possibility that VM also existed in epithelial neoplasm besides mesenchymal tumors. Our study demonstrates one more time that VM is not an individual event but a general phenomenon during tumor growth, being a functional microcirculation [26].

Tumors with VM have more capacity of invasion and metastasis [9, 20] and predict poor clinical outcome among tumor patients [8]. We posited from our previous study that VM was more likely to contribute to lymph node metastasis and was an unfavorable prognostic factor among LSCC patients (data not shown). Nasu et al.'s [27] and Sood et al.'s [4] study on melanoma cell lines also demonstrated that VM was linked to the aggressive tumor cell phenotype. The highly invasive melanoma cell line was successful in forming VM, while the low-invasive melanoma cell line failed to form VM. Further, our present study explored the mechanism of VM formation in HNSCC. Many factors are necessary in VM formation, including the microenvironment, the interaction between tumor cells and their surroundings, changing to endothelial genotype of the tumor cells, and extracellular matrix remodeling. EphA2 was first revealed by Hess et al. [11] which promoted VM formation through dephosphorylation of FAK and remodeling of extracellular matrix in VM formation in melanoma. Our study suggested that EphA2 may be a critical regulator for VM formation, cell migration, and invasion in HNSCC. However, unlike that in melanoma, our study focused on the changing of cell adhesion and cell plasticity regulated by EphA2 in terms of EMT.

Similar to VM, EMT is well correlated with invasion and lymph node metastasis [28]. It is a normal process in embryonic development in which epithelial cells transform into mesenchymal cells. It has likewise been reported to exist in HNSCC. Our study of* in vitro* and human samples demonstrated existence of VM related to the expression of EMT-related molecules. In addition, Maniotis et al.'s [3] analysis* in vitro* through cDNA microarray suggested that melanoma cells overexpressed both epithelial phenotype and mesenchymal phenotype correspondingly. The latter was a type VI collagen, a component of the extracellular matrix which was a major component of VM. It is interesting to speculate that highly aggressive epithelial tumor cells may likewise overexpress the mesenchymal phenotype through EMT during VM formation. Further, our study* in vitro* showed that EphA2 regulated both channel-like tubular formation and expression of EMT-related molecules. Changing of EMT is accompanied by the presence of VM. We conjectured from the above that EMT may be an alternative mechanism of VM formation in epithelial neoplasm. And further investigation is being done to explore new cotherapeutic targets for HNSCC treatment.

In summary, EphA2 plays a key role in VM formation in HNSCC through regulation of EMT. EMT may be an alternative mechanism of VM formation in HNSCC different from the mesenchymal tumor which depended more on remodeling of extracellular matrix. Those who are relying on conventional markers of tumor “vascularity” as prognostic markers and who are developing anticancer therapies by targeting angiogenesis should exercise caution concerning VM when interpreting their results. Further, combination of EphA2 and certain EMT-related molecules might be a potential therapeutic target for VM to control invasion and metastasis of HNSCC.

## Abbreviations

HNSCC: Head and neck squamous cell carcinoma
LSCC: Laryngeal squamous cell carcinoma
VM: Vasculogenic mimicry
EDV: Endothelium-dependent vessel
EMT: Epithelial-mesenchymal transition
EphA2: Ephrin type-A receptor 2
PCR: Polymerase chain reaction
SI: Staining index
PI: Positive index
DSG-3: Desmoglein-3.
